# Experience of Aging in the Ngäbe‑Buglé Community in Coto Brus, Costa Rica: A Qualitative Study

**DOI:** 10.5334/aogh.4544

**Published:** 2025-01-29

**Authors:** Melissa Rallo, Nicholas Leahy, Alexis Vetack, Hima Konduru, Shania Bailey, Lillianna Pedersen, Christine Wan, Wendel Mora, Virginia Rowthorn, Shailvi Gupta, Carlos Faerron Guzmán

**Affiliations:** 1 University of Maryland School of Medicine, Baltimore, MD, USA; 2 Centro Interamericano para la Salud Global (CISG), Puntarenas, Costa Rica; 3 Center for Global Engagement, University of Maryland Baltimore, Baltimore, MD, USA

**Keywords:** Social determinants of health, Indigenous health, Aging

## Abstract

*Introduction:* Indigenous communities grapple with unique challenges in the aging process, often encountering amplified barriers to healthcare and resources, possibly due to their remote locations and distinct cultural backgrounds. Limited research exists on aging within Costa Rica’s Ngäbe‑Buglé Indigenous community, particularly the La Casona community in Coto Brus. This study explores the aging experience of the Ngäbe‑Buglé community in La Casona, aiming to identify challenges and potential resources to enhance the quality of life and aging experience for elderly community members.

*Methods:* This qualitative study utilized semi‑structured interviews to collect data from 14 elderly participants (6 female, 8 male), aged 52–90 years, living in the La Casona community. Participants were selected through purposeful snowball sampling techniques, and individual interviews were conducted at their residences. Interviews were conducted with assistance from an interpreter and lasted approximately 30–60 minutes. Thematic analysis was used to examine participants’ responses to understand their experiences with aging.

*Results:* Three primary themes emerged: economic difficulties, insufficient social support, and cultural aspects related to La Casona. Among the three primary themes were a total of eight subcategories. Economic challenges encompassed financial constraints and food insecurity, housing and infrastructure needs, and difficulties in accessing healthcare. Insufficient social support was evident through heavy reliance on family, limited community aid, and an absence of engaging activities. Cultural aspects highlighted the community’s deep connection to nature and concerns about the fading cultural heritage among younger generations. These themes collectively contribute to the challenges confronted by elderly adults in the Ngäbe‑Buglé community.

*Conclusions:* Improving healthcare access, enhancing social interactions, and preserving cultural heritage are essential when it comes to improving the aging experience in La Casona. The following participant discussions provide insight into public health interventions. Addressing these issues will require governmental support and policy changes aimed at uplifting the Ngäbe‑Buglé community.

## 1. Introduction

**Figure 1:**
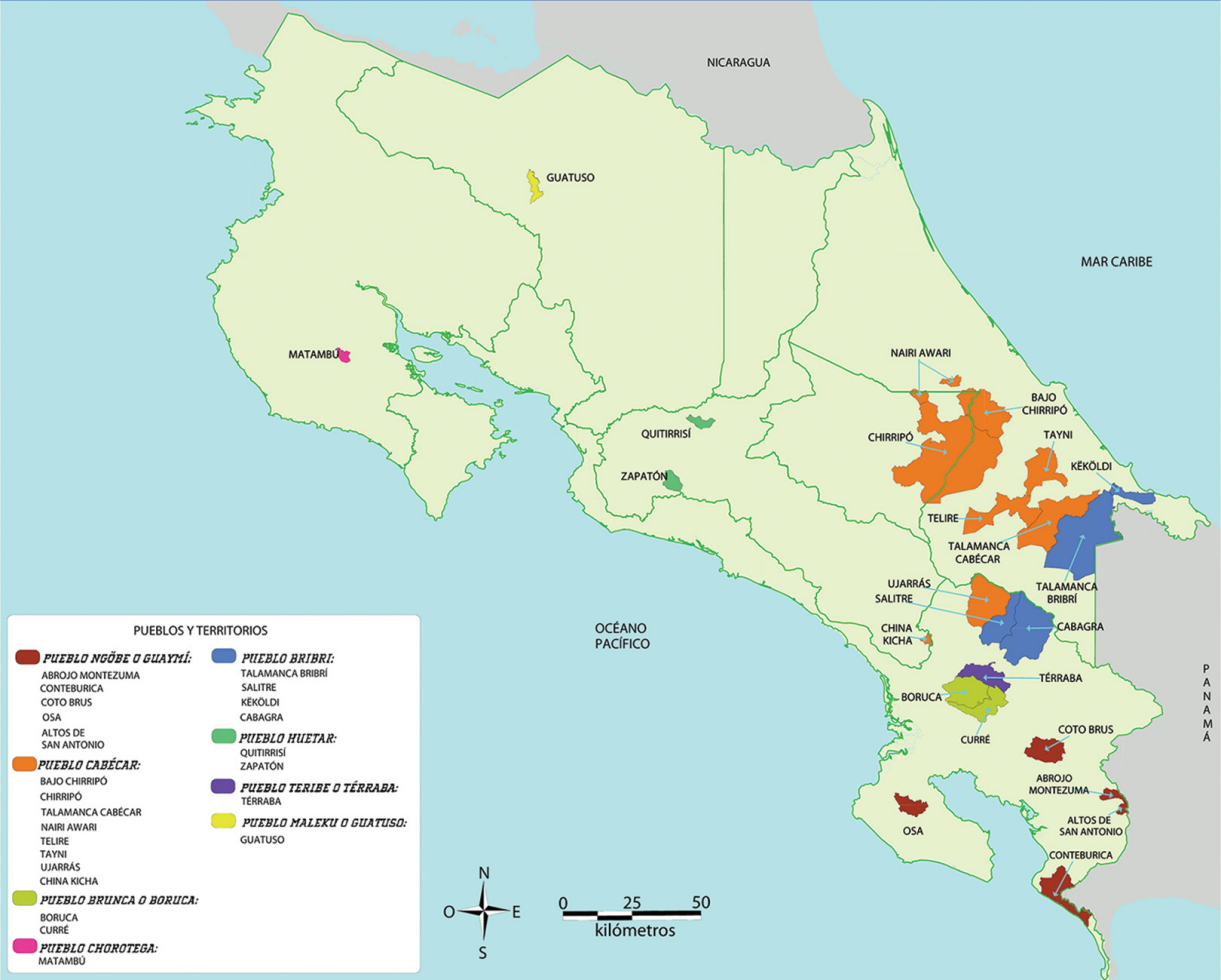
A detailed map of Costa Rica highlighting the locations of major Indigenous communities [1].

Costa Rica boasts a diverse Indigenous population, comprising eight distinct groups: the Huetar, Maleku, Bribri, Cabécar, Brunka, Ngäbe‑Buglé, Térraba, and Chorotega (Figure 1) [1]. These groups consist of 104,143 individuals, making up 2.4% of the total Costa Rican population [2]. Representing one of the eight unique communities, the Ngäbe‑Buglé people reside in Panama and Costa Rica, with ~250,000 residents throughout both countries [3]. In Costa Rica, the Ngäbe‑Buglé have five Indigenous territories: Abrojo Montezuma, Osa (Alto Laguna), Conte Burica, Altos de San Antonio, and Coto Brus [1]. These territories, established between 1980 and 2000 by federal decree, are relatively recent developments [4]. Notably, Indigenous communities constitute the majority of the workforce in Costa Rica’s coffee industry, highlighting their significant contribution to the nation’s economy [5]. The La Casona community, with 1,612 residents, is the most populous of the Ngäbe‑Buglé communities in Costa Rica, and is the focus of the present study [4].

Official recognition of the Ngäbe‑Buglé population took decades, culminating in a 2019 executive order that protected their right to nationality, addressing the previous lack of formal recognition of citizenship in both Costa Rica and Panama [6]. Despite recognition, continued issues over land ownership persist with 38% of Indigenous land in Costa Rica owned by non‑Indigenous people [6]. This represents an ongoing public policy issue, although a positive ruling by the Costa Rican Supreme Court reaffirmed Indigenous rights to their lands in October 2022 [7].

Additionally, 87% of the Ngäbe‑Buglé population falls below the poverty line, a stark contrast to the national average of 20% [3]. Indigenous communities also face marginalization through education disparities, with Indigenous children receiving an average of 3.6 years of schooling, resulting in a 70% literacy rate compared with 98% nationally [8, 9].

Globally, many nations are confronting the rapid aging of their populations. Although not a unique concern, Costa Rica is on the precipice of a demographic transition, with 11% of its population aged 65 years and older [9]. This trend is expected to rise to 20% by 2050 [4]. In Costa Rica, as in several other Latin American countries, elderly family members are often cared for by younger relatives within intergenerational households [10]. While the overall national life expectancy is high at 80.8 years, individuals residing in urban areas report better physical and mental health, as well as a higher health‑related quality of life, compared with their rural counterparts [11, 12]. It is important to note the contrast in aging experiences in Costa Rica between urban and rural areas, where most Indigenous communities reside.

Previous reviews of aging in global Indigenous communities emphasize social support, access to health resources, strong community bonds, and the ability to “age in place”: an elder’s preference to age at home, surrounded by family [13–16]. Additional studies noted a scarcity of resources for social interaction, and limited physical activity can further compound social isolation, leading to worsening experiences with aging [17, 18]. Although some initiatives to investigate aging within this Indigenous community exist, these studies are not well‑documented. The present study aims to investigate the main themes of aging and identify the needs of elderly adults in La Casona.

## 2. Materials and Methods

The present study employed a descriptive approach to describe perceptions of aging for the group of individuals that were interviewed in the La Casona community, without a pre‑determined hypothesis [19]. Descriptive studies aim to document characteristics or phenomena as they occur, without testing relationships or causality between variables [19]. The use of this approach was well suited to the study’s objective of understanding the community’s challenges and perspectives on aging, as it allowed for a detailed account of participants’ lived experiences. By focusing on predefined themes such as economic difficulties and cultural heritage, the study captured valuable qualitative insights.

### 2.1 Participants and recruitment

Participants were recruited from the Ngäbe‑Buglé community using the local Equipo Básico de Atención Integral en Salud (EBAIS) in the La Casona community. EBAIS sites are government‑funded primary care clinics for individuals throughout Costa Rica. Initial contact with the central EBAIS in La Casona was made through Dr. Pablo Ortiz, a community leader through the nonprofit Hands for Health who has a longstanding relationship with the La Casona community. Inclusion criteria included being a member of the Ngäbe‑Buglé community and being 50 years of age or older. Exclusion criteria included the presence of severe cognitive impairment (including dementia) or any impairment limiting social or daily function as recognized by family or neighbors. Purposeful sampling was initially employed to select participants who met our inclusion criteria. After each interview, a snowball sampling technique was implemented, where current participants were asked to recommend other elderly community members in the area who could also be interviewed. These techniques have been utilized and validated in qualitative studies of smaller communities [20, 21].

### 2.2 Ethical considerations

The study was approved by the institutional review board (IRB) at the University of Maryland (HP‑00105818) and by Costa Rica’s Comité Ético Científico Fundación Inciensa (CEC‑FUNIN; approved in session FIDE‑CEC‑099‑2023). Interviews were only conducted after participants were informed of study details and goals and agreed to participateand informed consent was given. The study was confidential, and interviews and data were only shared with research team members.

### 2.3 Data collection

Data collection took place over 4 weeks in June 2023. Prior to each interview, the study’s purpose was explained, and informed consent was obtained. The research team conducted interviews in Spanish, but a Ngäbe‑Buglé interpreter was present translating the conversation into Ngäbere, the community’s native language, if clarification was needed. Participants were informed they could stop the interview at any time should they feel uncomfortable or not wish to continue. Participants were asked beforehand whether the interview could be recorded. After obtaining informed consent, semi‑structured interviews were conducted with participants, lasting 30–60 minutes. Interviews were recorded and transcribed and took place at the participant’s own residence or family member’s home.

Predetermined questions were asked to all participants, informed by previous research on aging within other Indigenous communities [15, 22]. These questions focused on challenges related to social determinants of health, including infrastructure, healthcare access, nutrition, and the impacts of colonization [15]. Additionally, social isolation and community support questions were included, as prior studies have determined that isolation itself can lead to increased emotional and physical vulnerabilities [17]. Overall, questions focused on the perceptions of aging, local healthcare experiences, social support networks, and suggestions for improving the health and aging process in La Casona (Appendix 1).

### 2.4 Data analysis

Recorded interviews were fully transcribed and translated into English using Sonix.ai software, with edits made as needed by the research team. The interviewer team self‑translated when Sonix.ai was inaccurate. The transcripts were then manually coded using principles of grounded theory, a qualitative approach that emphasizes generating themes and insights directly from the data rather than relying on pre‑existing hypotheses [23]. Grounded theory involves iterative coding and comparison to uncover patterns and build a framework that reflects participants’ lived experiences.

Initial codes were generated following the five general categories outlined in the interview guide (Appendix 1). As data collection progressed, codes were refined and updated continuously, incorporating new insights from subsequent interviews. The research team, consisting of 10 members, independently reviewed and coded the transcripts to ensure reliability through cross‑checking and collaboration [24]. Final codes were grouped into three overarching themes identified among participants: economic difficulties, insufficient social support, and cultural aspects related to La Casona. This approach allowed for a comprehensive exploration of the data while mitigating individual bias, ensuring the findings were both nuanced and reflective of the Ngäbe‑Buglé community’s unique context.

### 2.5 Positionality

It is pertinent to acknowledge that none of the researchers are from the Ngäbe‑Buglé community, which may influence multiple aspects of this study [25]. The investigation team included members from the United States and Costa Rica, specifically from San Jose. Contact with the La Casona community was established through decades of relationship building by the nonprofit Hands for Health. The benefits of outsider status—of not belonging to the community of study—versus insider status—belonging to the community of study—have long been debated in research best practices. However, neither status is fixed, and both provide benefits as well as biases in research design [26].

## 3. Results

In all, 14 participants were interviewed (6 female, 8 male). They were 52–90 years old with education levels ranging from none (10 participants) to primary school completion (6th grade; Table 1).

**Table 1:** Demographic characteristics of participants.

PARTICIPANT NUMBER	SEX ASSIGNED AT BIRTH	AGE (YEARS)	EDUCATION LEVEL	CIVIL STATUS	OCCUPATION
1	Female	Unknown, ~78*	None	Married	Domestic work
2	Male	79	2nd grade	Married	Sells artisanal products
3	Male	52	None	Married	Agriculture
4	Male	90	None	Widowed	Retired
5	Male	Unknown, ~76*	4th grade	Single	Retired
6	Female	Unknown, ~75*	None	Widowed	Sells artisanal products, domestic work
7	Female	59	6th grade	Married	Retired, domestic work
8	Male	67	6th grade	Married	Agriculture
9	Female	Unknown, ~77*	None	Single	Sells artisanal products
10	Male	83	None	Single	Retired
11	Male	90	None	Married	Retired
12	Male	72	None	Married	Retired
13	Female	72	None	Single	Domestic work
14	Female	59	None	Married	Sells artisanal products

*Some participants were unsure of their exact age. Age estimates were given by participants as interviews were conducted.

Following questions derived from the interview guide (Appendix 1), three themes emerged along with eight subcategories (Table 2); these were analyzed using grounded theory. Many participants directly mentioned economic difficulties, lack of social support, and specifics of aging in La Casona.

**Table 2:** Themes and subcategories.

THEMES	SUBCATEGORIES
Economic difficulties	Funds
	Infrastructure
	Healthcare access
Insufficient social support	Reliance on family
	Little community support
	Limited activities and engagement
Cultural aspects related to La Casona	Importance of nature
	Generational gap

### 3.1 Economic difficulties

This theme comprises a variety of economic difficulties expressed by members of the community, including funding, better housing and infrastructure, and healthcare access.

#### 3.1.1 Economic difficulties: funds

When questioned about what could improve aging in the community (Appendix 1, question 5a), multiple participants expressed the need for increased financial assistance. Participants lived almost entirely off their pension provided by the government of Costa Rica, which becomes available to citizens at 65 years of age. The pension was noted to not be livable by most and is often used to support additional family members, including grandchildren.

*We need everything. Things (in my house) are broken. I need furniture, a washer, things for our houses… this will help older people in my community. We need food, we need money. Everything comes down to money. (P1)*

*Give more pensions. We have to waste it all with electricity, water and food. Then there is nothing. (P7)*

*I can’t walk. I am sitting in my house all the time. I need more help, more of a pension, more help with my house. The pension doesn’t cover everything… we don’t have money to take care of older people well. (P11)*

While pensions may support some elderly adults, not all individuals are physically able to travel and retrieve their pension. A family member must then get the funds for them, leaving some individuals vulnerable to financial exploitation.

*If you cannot walk, someone else has to go get their pension. A lot of times the people that go to take it out for their family members take some money from the older person. (P14)*

Participants noted that this, coupled with small pension amounts, can create challenges for larger families in the Indigenous community, where some may need to prioritize grandchildren and great‑grandchildren over elderly adults.

*Everyone is preoccupied with themselves because sometimes there is no food, no money. There’s a lot of grandkids, so older people are a bad investment for the family’s money… all the money goes to the grandkids. (P8)*

*People only think about the kids and grandkids, not us. (P9)*

With government pensions as the main monetary support for elderly adults in La Casona, individuals commented on food insecurity, especially at the end of the month, following questioning on challenges of aging in the community (Appendix 1, question set 1).

*Food and resources, no one brings it to me. I cannot work, so it’s hard… younger people have resources because they work, but older people don’t. If you don’t do anything, you don’t get anything. I deserve more resources. The last week of the month, my pension runs out. I think how will I eat? I pick bananas or cassava to eat that week. (P5)*

*If I had more money, I would buy more food, vitamins, medicine… but especially food. (P14).*

#### 3.1.2 Economic difficulties: infrastructure

Multiple participants mentioned the need for better infrastructure in their community when questioned about challenges of aging and suggested improvements (Appendix 1, question set 1, question set 5), especially in the form of individual home improvements.

*Improvements in the home and bigger homes to fit our families. We need more infrastructure. (P3)*

*If I had more money, I would use cement in my house to make my floors better and fix my kitchen. I also would have better places to sit, a good mattress. (P6)*

While not just an issue for elderly adults, participants mentioned how a lack of infrastructure affected their ability to move and walk. Given the proportion of dirt roads in the community, participants noted that the terrain can become dangerous, especially during the rainy season. Elderly community members highlighted that this impacts their daily lives, limiting their ability to seek quality social interactions outside the home.

*I want to improve things for me to walk. Maybe so things don’t get so wet. It’s hard (for me) to go into the house. That is how I fell… it is dangerous. (P1)*

*It’s hard to get around. If you can’t walk, then there is nothing. You can’t use a wheelchair anywhere here. (P6)*

*When it rains, people cannot walk here a lot. (P7)*

Finally, participants identified a need for a recreational gathering space that is specific for elderly community members to improve the aging process (Appendix 1, question 5a).

*A home for older people. It’s hard for people who live far away to get to the center. With the home, they won’t have to walk as far, and you can reach the center by car. Ambulances can reach it. (P7)*

*A place to meet, and a nursing home. Place to give out food to older people and hold events, birthday parties. Maybe even schedule a tour, like to San Isidro. People can walk around, and everyone comes back with a different, better mentality. To have a place of our own, or even minibuses to take older people outside to a new place and walk around. This would help us a lot. (P8)*

#### 3.1.3 Economic difficulties: healthcare access

All formalized healthcare in La Casona goes through the EBAIS system. While the clinic is centrally located, participants noted that it is difficult to access from houses located on the outskirts of La Casona when questioned about healthcare improvement (Appendix 1, question set 2). Some community members commute a few hours to access the EBAIS clinic on foot.

*If I am really sick, I go to EBAIS. If it’s really bad, I go to San Vito, but that costs money and is far away from my family. (P6)*

*A lot of people go (to the EBAIS) and walk hours, and they get there and it’s full. We need more healthcare, more doctors. (P10)*

Additionally, the EBAIS only offers primary care services. All special services need referrals and are often located in downtown San Vito, a 45–60 minute drive from La Casona.

*Access to healthcare is bad for others who live farther away. More access to healthcare services is important to me, this would improve my life a lot. Here, if you need more tailored healthcare, you have to go outside, and then also come back. That’s why transport is important to me too. Most people don’t have the money to go there and back…*

*It’s hard for them (EBAIS) to attend to us. There’s not enough. And also, they can’t really help us that much, you have to go to specialists. What we need is another doctor. We need another doctor, because there’s so many people. And the poor doctor, even the best doctor, doesn’t have a chance, there are too many people who have different issues. (P12)*

### 3.2 Insufficient social support

This theme comprises aspects that contribute to a lack of social support in the community. Participants expressed family support as a primary means of social support while noting a lack of formal programs, dedicated gathering places, or social meetings for elderly members of the community.

#### 3.2.1 Insufficient social support: reliance on family

Elderly members of the Ngäbe‑Buglé community rely entirely on their families for care, as no formal community support systems, such as an elderly home, exist. This reliance leads to significant variability in the level of care provided, depending on the family’s circumstances and capacity.

*The family takes care of older adults in the house. If you don’t have family, the community helps some. But the family is the main support system. (P2)*

*When the elderly can no longer walk, they need family to accompany them, to take care of them, to cook and to get food for them. (P10)*

In contrast to grandkids contributing to the financial burden on elderly adults as discussed in 3.1.1, when questioned about social support (Appendix 1, question set 3), participants spoke positively about their families taking care of them and highlighted the younger generation’s role.

*Family takes care of everyone. I took care of my kids, so now that I am older, they have to repay that by taking care of me. (P7)*

*My grandkids or kids take care of me. They live close to me and I am like a king, because they have to bring me everything… I can’t walk. (P11)*

#### 3.2.2 Insufficient social support: lack of community support

The lack of formal support such as nursing homes, community aids and social workers, and caretakers may create disparities, as some families provide attentive care, while others may disregard their older members. Without a community‑based safety net, elderly individuals without familiar support may face significant challenges in meeting their basic needs and maintaining social connections. The elderly adults in La Casona below discussed feeling neglected by the community, with little personal autonomy (Appendix 1, question set 3).

*There is no dedicated attention to older people, like saying what do you want to eat today or even if you do want to eat. What’s given to you is given to you and that’s that…*

*Community support doesn’t exist. People have not yet understood that we have to take care of the elderly. Every family takes care of their eldest as best they can. (P8)*

*The community doesn’t support its older members. The students and younger people only care about themselves. They don’t care if the older people are suffering or not, they just don’t care. They don’t care for older people, they think about their futures. (P10)*

#### 3.2.3 Insufficient social support: limited activities and engagement

A large concern of elderly adults in La Casona was the lack of available activities and social gatherings.

*We don’t do anything; we are all in our house and stay in our house because we cannot do anything. (P4)*

*In La Casona, older people do nothing… There is no one to talk to all day, that also makes me sad. (P5)*

*We don’t do anything. I can’t do anything really anymore. So, we just stay inside sitting. (P14)*

Furthermore, elderly adults noted having no formal gathering places or events for social interaction. As mentioned in section 3.1.2, there is a great need for further infrastructure such as roads, gathering places, and larger homes for elderly adults to socialize. This was a priority for many of our participants who expressed feeling neglected and bored in their houses all day.

*There are no activities for older people in La Casona. You can take the bus to San Vito for more things. Sometimes I do exercise or dance there in San Vito. Here there is nothing. (P6)*

*I want a place to do activities and talk to other older people. I am bored. (P8)*

*Older people are only in the house. Sometimes they make artisanal crafts but nothing else… The community doesn’t think about older people, only themselves. (P9)*

### 3.3 Cultural aspects related to la casona

The last theme highlights how cultural aspects contribute to the experience of aging. Participants noted cultural ties to nature while also mentioning the presence of a generational gap in the community.

#### 3.3.1 Cultural aspects related to la casona: importance of nature

Participants expressed a deep appreciation of the beauty and significance of La Casona, the ancestral land that the Indigenous community in Coto Brus has lived on for generations. Nature plays a vital role in the community, with both appreciation and respect, particularly in relation to traditional medicine.

*It’s all okay, the nature is beautiful. (P2)*

*The land is beautiful. There is good food and the water is potable. (P5)*

*To me, traditional medicine is better. (P6)*

*The lands are free. That God left the lands free for us. That the earth is sacred. When we die, we go under the earth and that’s why we believe in the earth and that’s why we are respectful. And that is the land. Like a mother, it gives water, food. It gives everything, everything. When we die, we go back to it. (P10)*

When asked about support for the Indigenous community from outsiders (Appendix 1, question 5b), one participant emphasized the importance of historical context. They pointed out that Spanish colonialism and subsequent actions by the Costa Rican government led to the loss of Indigenous lands, despite the community having lived there for centuries. They suggested that this historical context justifies reparations and the provision of greater government aid, which multiple participants echoed.

*We know the history of the land, of medicine. We know how we used to live, before Christopher Columbus. There were millions of Indigenous people from Argentina all the way to Canada. It was all mountains, full of livestock that didn’t destroy the land, because to us, the land is sacred. You could live until 100, still walking well. And then the Europeans arrived. They destroyed everything…*

*I very much agree that organizations, companies, foreigners should support the Indigenous people, because the government of the country is indebted to us… they exploited our land... they exploited the Indigenous people. They are indebted for killing the land, and thus the lives of Indigenous people. (P10)*

*External organizations should give us support. Only the pension really helps. They should give us more things like mattresses or furniture for our house. (P5)*

*They should help us with what they can—food, clothes, everything. They should help us because they see the poverty that the Indigenous live in. (P13)*

*I hope outside organizations would support us. We don’t have any money. (P14)*

#### 3.3.2 Cultural aspects related to la casona: generational gap

When questioned about challenges and cultural aspects of aging (Appendix 1, question set 1, question set 5), elderly adults in La Casona expressed a sentiment of cultural erosion within the younger generation. This perception arises not solely from the impact of contemporary trends and pervasive Western influences but also from their belief that the younger cohort exhibits a disinterest in embracing Indigenous cultural heritage. This generational gap is significant, as it may impact the aging experience, particularly if younger family members are the primary caregivers for the elderly, as highlighted above. For the older generation, cultural heritage is a key source of identity and value in aging, providing opportunities to share traditions and foster a sense of community.

*Younger people think our culture is set in its ways. They don’t appreciate it the way we do. (P3)*

*There are a lot of beautiful things about our culture that are lost on younger people. Culture, greater ways of thinking, greater ways of living, have already been lost because the larger society is invading it… the Indigenous cultural identity, like education and language, is being lost…*

*If I had more money, I would write a book about our culture, so it is not lost on the younger generation. The Ngäbes have a very different history than common history. There has to be someone from here, a student from our school, an Indigenous student themself, that can write their own book that stays in the community. (P8)*

*The older people maintain the culture, but the younger people don’t want it. (P13)*

## 4. Discussion

Aging within the Indigenous community of Coto Brus is a multifaceted experience, encompassing both challenging and positive aspects. This study identified various resources needed by elderly Ngäbe‑Buglé adults.

Participants noted profound economic needs, including funds and food insecurity, infrastructure, and healthcare. These challenges have been previously noted to have negative impacts on aging [15]. In La Casona, resources intended for the elderly were often prioritized to aid children and grandchildren. Additionally, a demand for enhanced infrastructure became evident at both the familial and community levels. This pertained to the need for resilient, flood‑resistant dwellings with improved amenities and upgraded roads to ensure safer access to resources in the community. Participants also identified restricted access to healthcare due to the geographical isolation of the central EBAIS facility and its limitations, specifically its lack of specialized care and patient overload. This issue is exacerbated for older adults who may struggle to walk to the EBAIS without the help of a caregiver. This issue in the Ngäbe‑Buglé community is not unique, and has been identified in other Indigenous communities globally [22, 27].

The historical marginalization of Indigenous communities in Costa Rica, evident through high levels of poverty and lower education levels compared with the general population [3, 8, 9], contribute to limited economic resources and state aid. This marginalization partially stems from the idea of statelessness, or not belonging to any nation due to a lack of birth registration. Statelessness leads to a multitude of effects, including barriers to scholarships for students pursuing higher education [28].

Participants also identified insufficient social support for elderly adults, evident in the sense of heavily relying on family for caregiving and lack of formal activities among elders in the community. With the burden of elder care placed on family members, this can strain familial relationships and present challenges for elderly adults who do not have larger or supportive families. This strain can leave elders at risk for prolonged periods of solitude and inactivity within their homes, especially in the absence of community activities that can foster non‑kin relationships—a type of bond that has been established as a protective measure against social isolation, along with other physical and emotional vulnerabilities [17]. With this apparent need to strengthen social interaction between elderly adults outside the family, one solution lies in bolstering infrastructure (as mentioned mainly in the context of another theme), as mobility is crucial for accessing neighbors and resources in the community. Limited physical activity itself can also exacerbate loneliness and social isolation [18].

While the study identified many needs for the elderly community living in La Casona, general tenets of aging were discovered specifically in the context of the Ngäbe‑Buglé community. Participants discussed the sacredness of and protectiveness they feel over their ancestral land, underscoring a perspective in support of reparations, especially since executive decrees did not start returning land until the 1950s [3, 29]. Nature was also highlighted as playing a therapeutic role, with some participants opting for traditional medicine as a primary resource, prior to visiting the EBAIS. This finding could be influenced by age, with participants noting that most of the younger generation have shifted away from Indigenous practices. This is a finding that is not unique to the La Casona community [30]. Lastly, participants expressed concerns about the waning appreciation of Ngäbe‑Buglé culture, particularly the gradual decline of their native language. This apprehension resonates globally, as 90% of the world’s languages are predicted to be lost in the next 100 years, many of them Indigenous [31]. The erosion of cultural heritage has been partially counteracted through the inclusion of Indigenous teachings in local schools; nonetheless, the elders’ concerns regarding cultural loss remained palpable.

## 5. Conclusions

Opportunities exist for enhancing aging and healthcare in the Ngäbe‑Buglé community of Coto Brus, Costa Rica. Key areas for improvement relate to the overarching themes of the present study, including economic support, social support, and culture. The study recommends the following general areas of improvement:

1. **Increase access to resources for elderly adults, especially food and safe living environments.** Providing food directly to elderly adults can be a protective measure against inadequate pensions not covering basic resources or funds being allocated to younger family members. Additionally, efforts should be directed toward securing safe housing and essential furniture, which may improve quality of life and health outcomes [32].
2. **Improve community healthcare access through increasing the number of providers and establishing a system for community members to consult specialists when necessary.** This may include a transportation service for those requiring specialized care in San Vito town hospitals. Significant steps have already been taken to integrate Indigenous representation into the local hospital system, including a full‑time care coordinator from La Casona. Likewise, the incorporation of culturally sensitive birthing practices and hospital gowns reflects a positive shift toward inclusivity and culturally sensitive care practices. Increasing access to healthcare has the potential to improve care not only for elderly adults but also for people of all ages who may need specialized care.
3. **Promote social connectivity and cultural preservation.** Social gatherings tailored to elderly adults can provide opportunities to foster interactions outside of the family and address the social isolation identified in our study. Local leaders should thus consider developing communal meeting spaces and nursing homes as referenced by multiple participants. Additionally, this initiative not only addresses social isolation but also could bridge generational gaps and aid concerns regarding the preservation of cultural practices and language. These platforms could be utilized in the community for the exchange of knowledge and experiences, potentially documenting and preserving oral histories and accounts shared by elderly community members.

It is crucial to highlight that addressing these issues requires increased support from both the Costa Rican government and local authorities. As a community with a documented history of neglect and marginalization, systemic changes are required at the national level to uplift Indigenous communities not just in Coto Brus but throughout Costa Rica. Despite recent strides, such as ensuring formal birth registration and reaffirming Indigenous land rights, significant work remains ahead [7, 28]. When assessing the impact of underinvestment in Costa Rica’s Indigenous populations, it is essential to consider the negative impact on the elderly and the aging process.

## 6. Strength and Limitations

This study aimed to explore aging in the Indigenous community of Coto Brus, Costa Rica, with a focus on the Ngäbe‑Buglé community. Given the lack of prior literature related to aging in this community and other Indigenous territories as a whole, the researchers plan to disseminate findings from the Ngäbe‑Buglé elderly to local authorities, including the Coto Brus government, EBAIS, and public health entities. The researchers also hope that the present study will serve as a framework for how to employ a qualitative design to learn about marginalized communities.

Prior research has highlighted the importance of implementing an anti‑colonial framework in research to celebrate intangible aspects of Indigenous culture and share findings in a non‑appropriating manner [33]. In this way, we hope to amplify Indigenous voices, honor the unique aging processes of the Ngäbe‑Buglé community, and prompt change among local policymakers on the basis of these interview findings.

This qualitative study had some limitations. First, this study is not broadly generalizable and focused a deep understanding on aging in a single community. The participant sample, drawn exclusively from Ngäbe‑Buglé individuals in La Casona, potentially limits the transferability of results to other Indigenous communities in Costa Rica or Ngäbe‑Buglé individuals living elsewhere. Moreover, this study was conducted over a specific 4‑week period in June 2023, which may not capture seasonal variations or dynamic changes in the aging experience throughout the year. An extended data collection timeframe could provide a more comprehensive understanding of the community’s aging challenges and resources. Furthermore, recall and survivor bias may have affected the study’s validity and generalizability, as interview questions prompted reflection in participants, and all interviewees were over the age of 52 years. To protect against interview bias, the study design applied purposeful snowball sampling, pre‑determined interview guides (Appendix 1), and recording and transcribing interviews.

Additionally, language barriers may have influenced study findings. As most Ngäbe‑Buglé people do not speak Spanish natively or use it to communicate among themselves, expressing more complicated thoughts surrounding aging may have been difficult. The interviewers only spoke Spanish and English, limiting the conversation to not include Ngäbere. Thus, some information or personal experiences of interviewees may have been omitted. Some participants also required an interpreter to repeat interview questions in Ngäbere, which may have misconstrued the meaning. Despite measures to ensure accurate translations, such as having multiple team members review transcripts prior to translating, some variations in interpretation may have occurred.

Finally, the research team consisted of outsiders to the La Casona community, as discussed in Section 2.5: Positionality. Our outsider status may have introduced elements of mistrust and a lack of complete understanding of our interviewees’ lived experiences [34]. However, this status may have also allowed for a more objective research process through the ability of the research team to distance themselves from the community of study [34].

## Additional File

The additional file for this article can be found as follows:

Supplementary Appendix 1. Interview guide for understanding Indigenous perspectives on aging and health.
