# Supplementary material for: Experience of Aging in the Ngäbe‑Buglé Community in Coto Brus, Costa Rica: A Qualitative Study
Source: Ann Glob Health. 2025 Jan 29;91(1):8. doi: 10.5334/aogh.4544 (PMC11784519; doi:10.5334/aogh.4544)
Supplement: Supplementary Appendix 1. — Interview guide for understanding Indigenous perspectives on aging and health. [file agh-91-1-4544-s1.pdf]

Appendix 1: Interview guide for understanding Indigenous perspectives on aging and health

Introduction: We appreciate your willingness to participate in this interview. Your insights will greatly assist us in our project aimed at understanding the determinants of health of older adults in Coto Brus. All answers will be kept confidential and will be used for research purposes only.

Sociodemographic Profile	
1. Full Name: ________________________________	2: Age: ______________________
3. Address: __________________________________	4: Gender: ___________________
5: Occupation: _______________________________	6. Civil Status: ______________

1. **Perceptions of Aging**
    a. Can you describe what it means to grow old in your community?
    b. What are the positive aspects of aging in your community?
    c. Do you think that older individuals have challenges in your community? Can you give some examples?
2. **Health and Aging**
    a. How would you describe the overall health of older individuals in your community?
    b. What are the most common health problems affecting older people in your community?
    c. When you are not feeling well, where or with whom do you seek help?
3. **Support and Care**
    a. Who typically takes care of older individuals within your community?
    b. How does the community support its older members?
    c. Are there any services or resources you wish were available for older people in your community?
4. **Cultural and Spiritual Aspects**
    a. What are the roles of elders within your community?
    b. Are there specific cultural or spiritual beliefs in your community regarding aging?
    c. How do these beliefs influence the care and support provided to older individuals?
5. **Suggestions for Improvement**
    a. If you could change or improve anything about the aging experience in your community, what would it be?
    b. What role do you think outside organizations or institutions should play in supporting aging populations in your community?

Your time and insights are greatly appreciated. Your input will contribute significantly to our understanding of aging in your community and how we can best support its older members.
